# Supplementary material for: Reduction-Hypersensitive Podophyllotoxin Prodrug Self-Assembled Nanoparticles for Cancer Treatment
Source: Pharmaceutics. 2023 Feb 27;15(3):784. doi: 10.3390/pharmaceutics15030784 (PMC10058384; doi:10.3390/pharmaceutics15030784)
Supplement: Supplementary file 1 [file pharmaceutics-15-00784-s001.zip › pharmaceutics-2203449-supplementary.pdf]

Supplementary Materials

Reduction-Hypersensitive Podophyllotoxin Prodrug Self-Assembled Nanoparticles for Cancer Treatment

Xinhui Wang [†], Yuequan Wang [†], Jiaxin Yu, Qian Qiu, Rui Liao, Shenwu Zhang, Cong Luo [*]

Affiliations:

Department of Pharmaceutics, Wuya College of Innovation, Shenyang Pharmaceutical University, Shenyang 110016, China

† These authors contributed equally to this work.

*** Corresponding authors:**

Cong Luo, Ph.D.

Department of Pharmaceutics, Wuya College of Innovation, Shenyang Pharmaceutical University, Shenyang 110016, China

Tel: +86-24-23986321; Fax: +86-24-23986321

E-mail: luocong@syphu.edu.cn

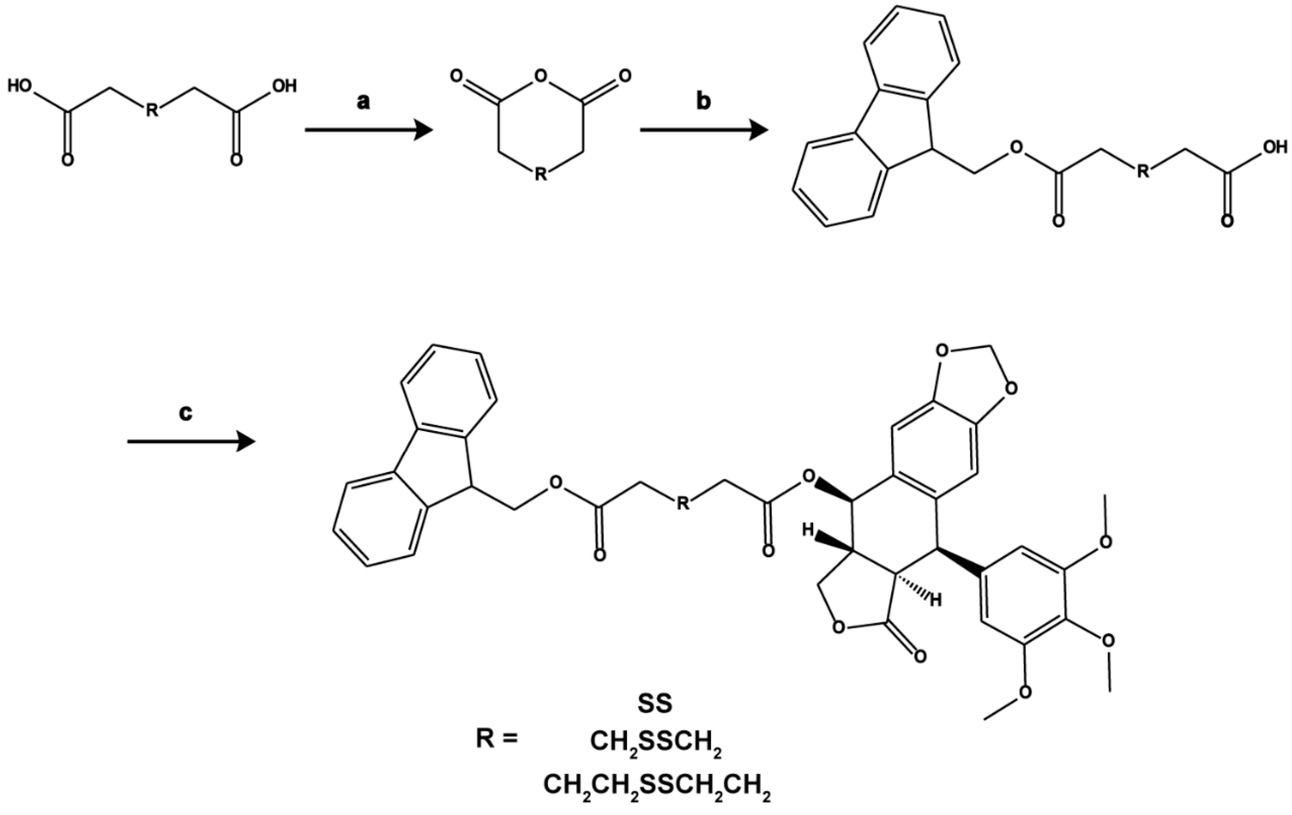

(a): acetic anhydride, 25˚C; (b): 9-fluorene methanol, DMAP, 25˚C; (c): EDCI, HoBt, DMAP, 0˚C; PPT, 25˚C.

Figure S1. Synthetic route of designed disulfide bond-bridged PPT prodrugs.

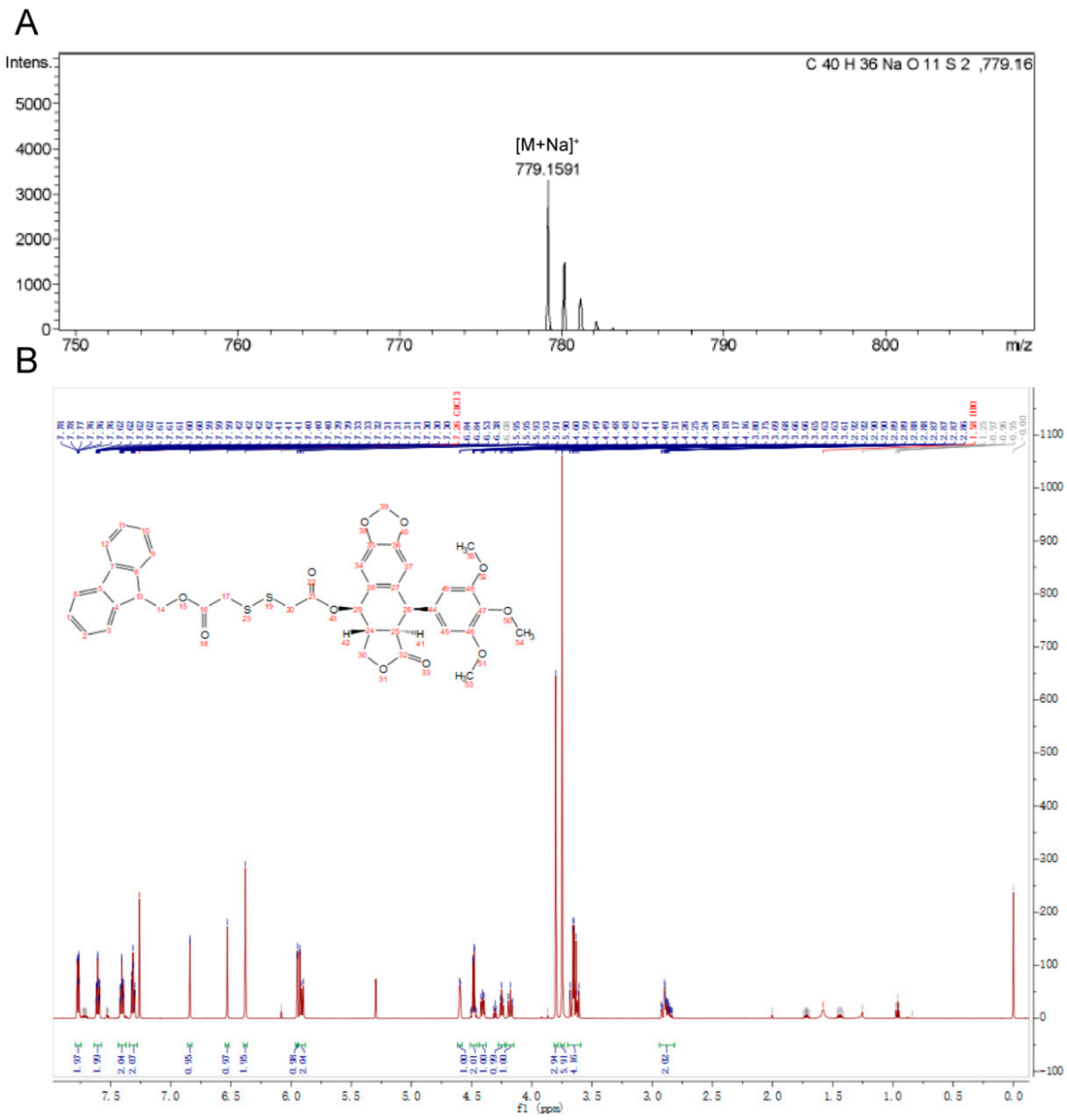

Figure S2. (A) MS and (B) ^{1}H NMR of FAP. $[M+Na]^{+}$=779.16, ^{1}H NMR (600 MHz, Chloroform-*d*) δ 7.77 (dt, *J* = 7.6, 0.9 Hz, 2H, 6, 12), 7.61 (tq, *J* = 7.5, 0.9 Hz, 2H, 1, 11), 7.41 (tq, *J* = 7.5, 0.9 Hz, 2H, 2, 10), 7.31 (tt, *J* = 7.4, 0.9 Hz, 2H, 3, 9, 34), 6.84 (d, *J* = 0.7 Hz, 1H, 37), 6.53 (s, 1H, 29), 6.38 (s, 2H, 45, 49), 5.95 (d, *J* = 1.4 Hz, 1H, 13), 5.94 – 5.88 (m, 2H, 39), 5.30 (s, 0H), 4.60 (d, *J* = 4.0 Hz, 1H, 26), 4.48 (dd, *J* = 7.2, 1.1 Hz, 2H, 14), 4.41 (dd, *J* = 9.2, 6.4 Hz, 1H, 42), 4.25 (t, *J* = 7.1 Hz, 1H, 30'), 4.21 – 4.15 (m, 1H, 30''), 3.80 (s, 2H, 17), 3.75 (s, 6H, 53, 55), 3.70 – 3.59 (m, 4H, 20', 54), 2.94 – 2.82 (m, 2H).

A

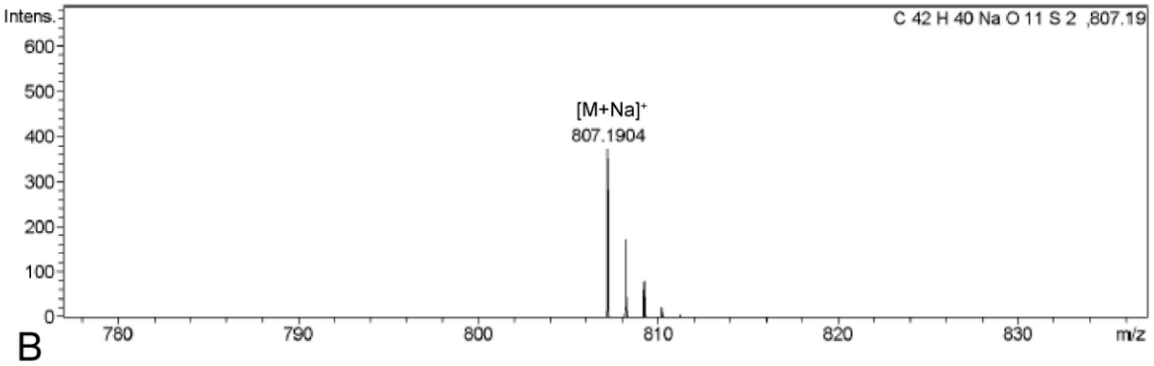

B

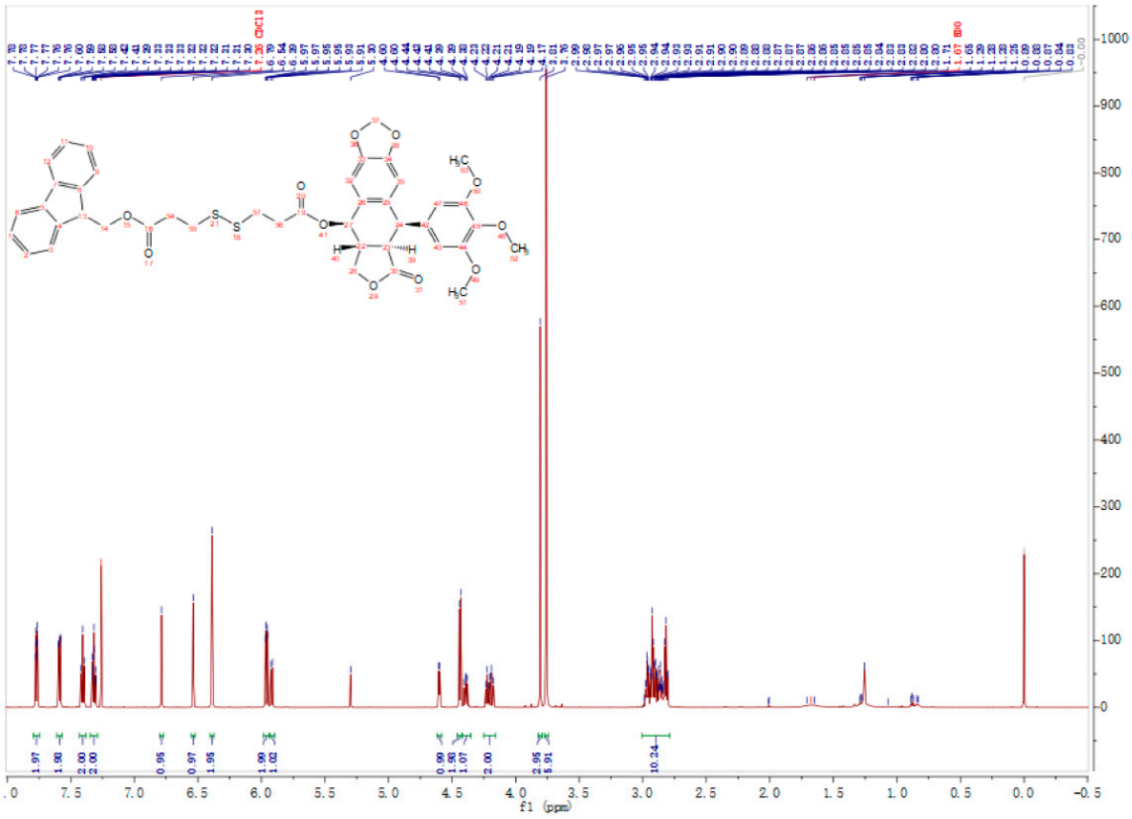

Figure S3. (A) MS and (B) ^{1}H NMR of FBP. $[M+Na]^{+}$=807.19, ^{1}H NMR (600 MHz, Chloroform-*d*) δ 7.77 (dt, *J* = 7.6, 0.9 Hz, 2H, 3, 9), 7.59 (dd, *J* = 7.5, 1.1 Hz, 2H, 6, 12), 7.41 (t, *J* = 7.5 Hz, 2H, 1, 11), 7.32 (tt, *J* = 7.4, 1.0 Hz, 2H, 2, 10), 6.79 (s, 1H, 35), 6.54 (s, 1H, 32), 6.39 (s, 2H, 37', 43, 47), 5.96 (dd, *J* = 9.5, 1.4 Hz, 2H, 13, 27, 37''), 5.92 (d, *J* = 9.1 Hz, 1H, 27), 4.60 (d, *J* = 4.4 Hz, 1H, 24), 4.44 (d, *J* = 7.1 Hz, 2H, 14), 4.39 (dd, *J* = 9.3, 6.9 Hz, 1H, 40), 4.25 – 4.16 (m, 2H, 28), 3.81 (s, 3H, 52), 3.76 (s, 6H, 51, 53), 3.00 – 2.79 (m, 10H, 39, 54, 55, 56, 57), 1.25 (s, 1H,).

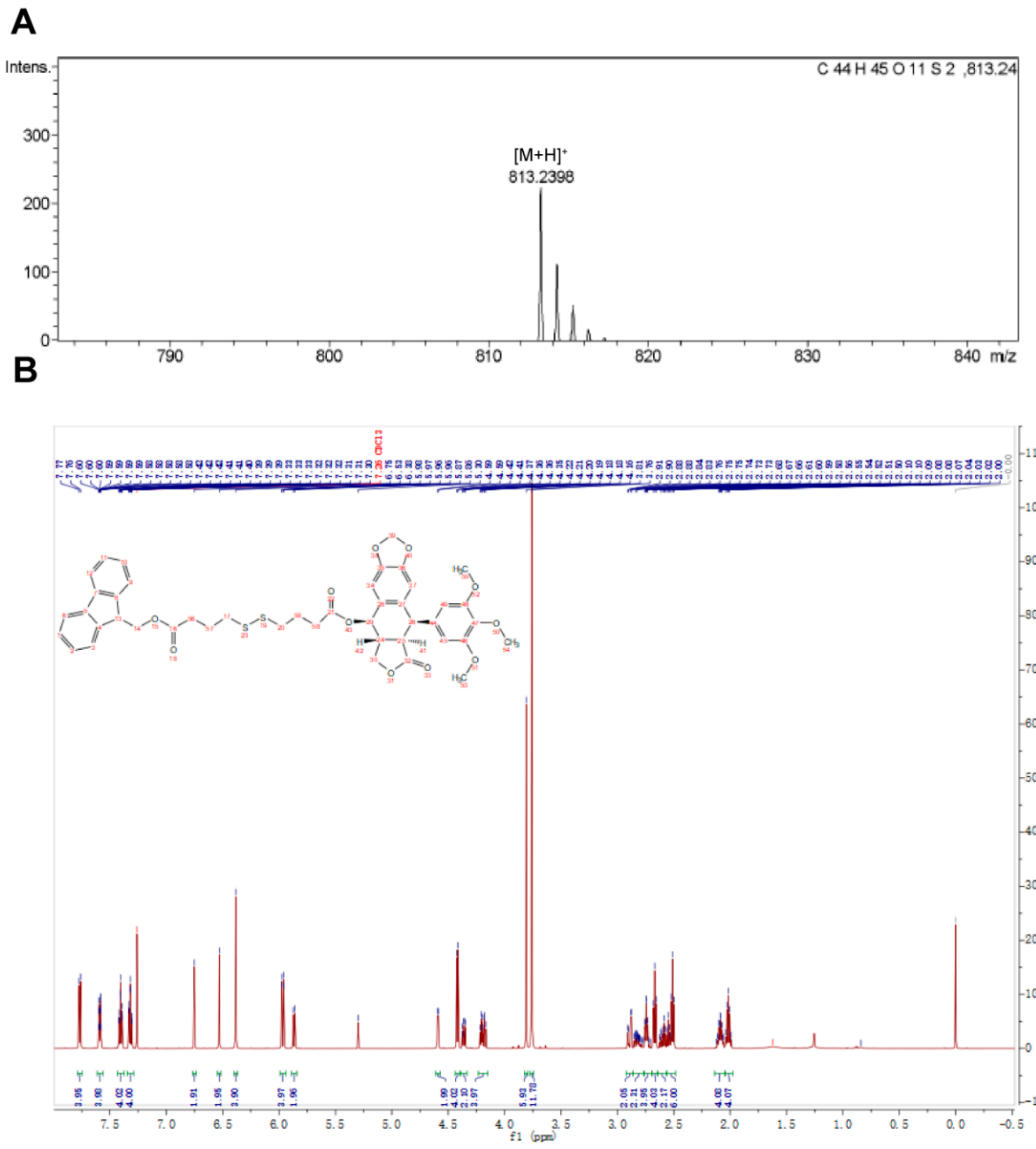

Figure S4. (A) MS and (B) ^{1}H NMR of FGP. $[M+H]^{+}$=813.24, ^{1}H NMR (600 MHz, Chloroform-*d*) δ 7.77 (d, *J* = 7.5 Hz, 2H, 6, 12), 7.59 (ddq, *J* = 7.5, 1.8, 0.9 Hz, 2H, 3, 9), 7.41 (tt, *J* = 7.5, 0.9 Hz, 2H, 1, 11), 7.32 (tt, *J* = 7.5, 0.8 Hz, 2H, 2, 10), 6.75 (s, 1H, 37), 6.53 (s, 1H, 34), 6.38 (s, 2H, 45, 49), 5.97 (dd, *J* = 10.9, 1.4 Hz, 3H, 13, 39), 5.87 (d, *J* = 9.1 Hz, 1H, 29), 4.59 (d, *J* = 4.4 Hz, 1H, 26), 4.42 (d, *J* = 7.0 Hz, 2H, 14), 4.36 (dd, *J* = 9.3, 6.9 Hz, 1H, 42), 4.23 – 4.15 (m, 2H, 30), 3.81 (s, 3H, 54), 3.76 (s, 6H, 53, 55), 2.89 (dd, *J* = 14.5, 4.4 Hz, 2H, 58', 58"), 2.86 – 2.76 (m, 1H, 41), 2.74 (td, *J* = 6.9, 2.1 Hz, 2H, 41, 58"), 2.67 (t, *J* = 7.1 Hz, 2H, 56), 2.60 (dt, *J* = 16.5, 7.3 Hz, 2H, 17), 2.53 (dt, *J* = 22.9, 7.2 Hz, 3H, 17, 20'), 2.09 (pd, *J* = 7.1, 2.6 Hz, 2H, 59), 2.02 (p, *J* = 7.2 Hz, 2H, 57).

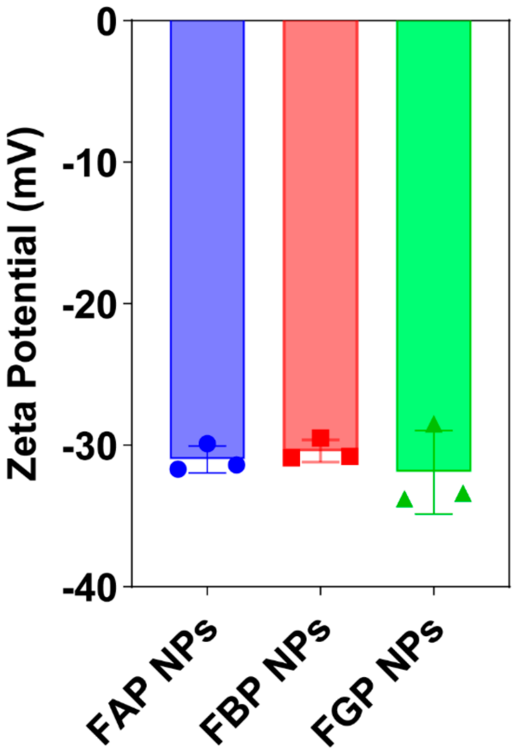

Figure S5. Zeta potential of FAP NPs, FBP NPs and FGP NPs.

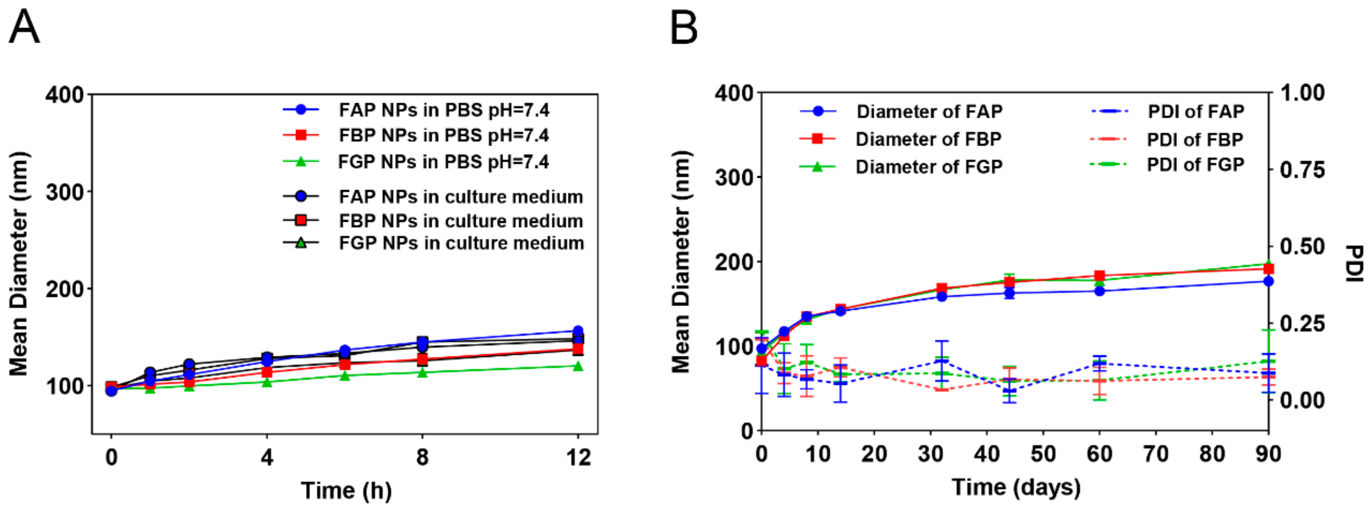

Figure S6. (A) Stabilities of prodrug NPs in PBS and RPMI 1640 culture medium (with 10% FBS). (B) Particle size and PDI of prodrug NPs in 90 days.

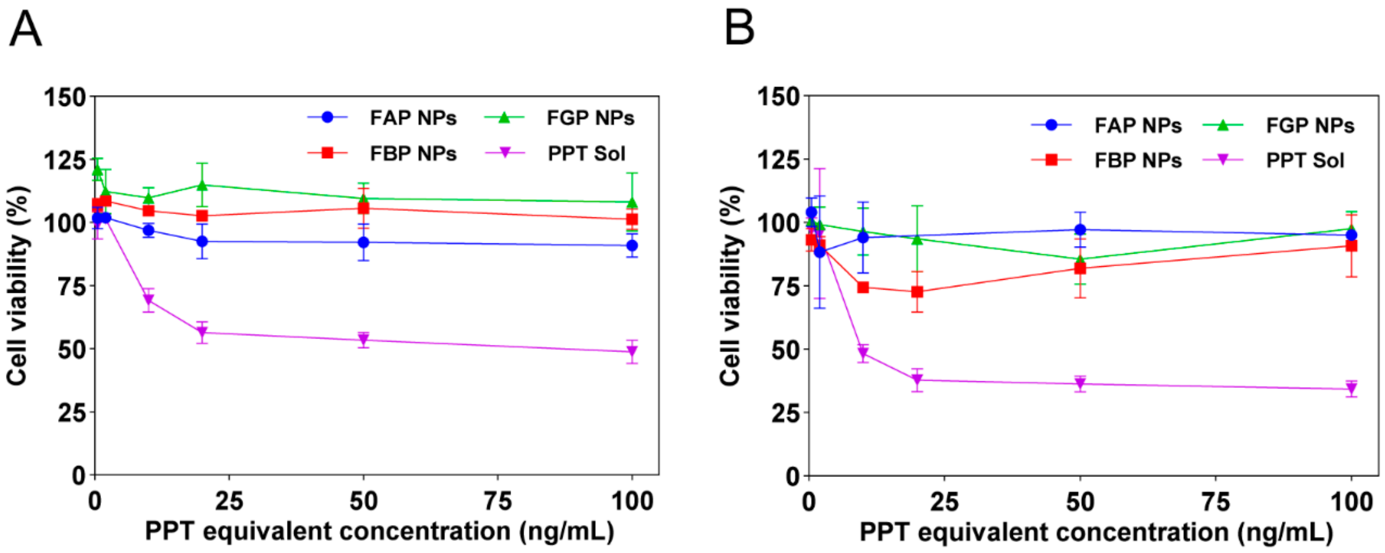

Figure S7. Viability of 3T3 cells after treated with various concentrations of PPT and prodrug NPs for (A) 48 h and (B) 72 h. Data are presented as mean ± SD (n = 3).

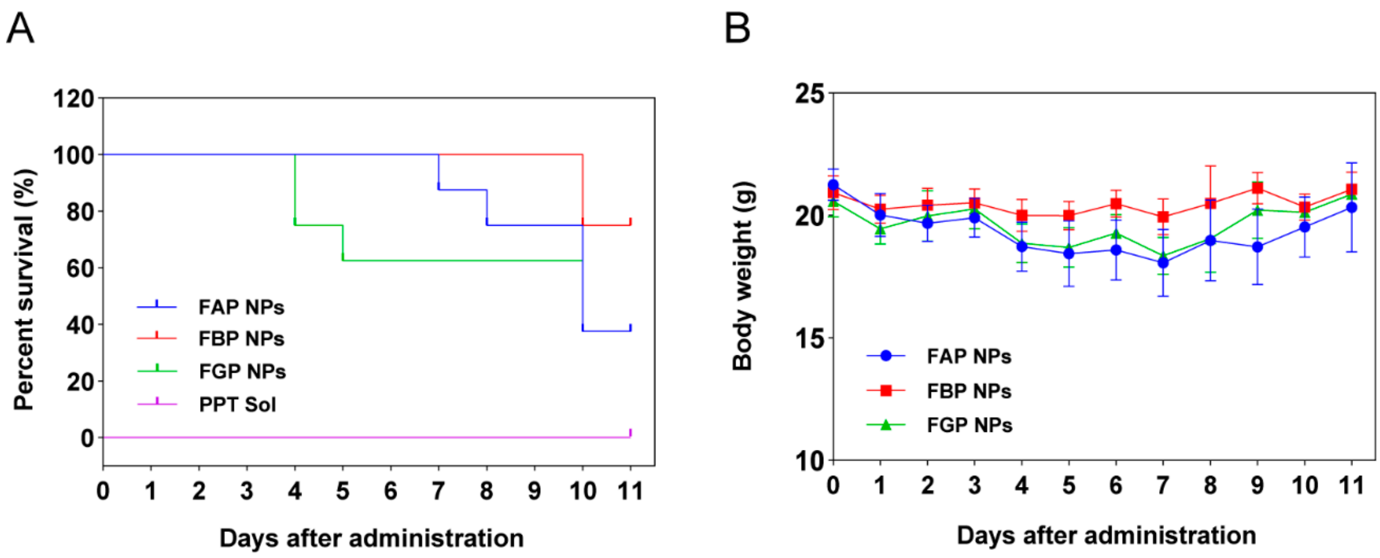

Figure S8. (A) Survivorship curve and (B) body weight changes following i.v. injection of prodrug NPs at PPT-equivalent dose in 30 mg/kg (n = 8).

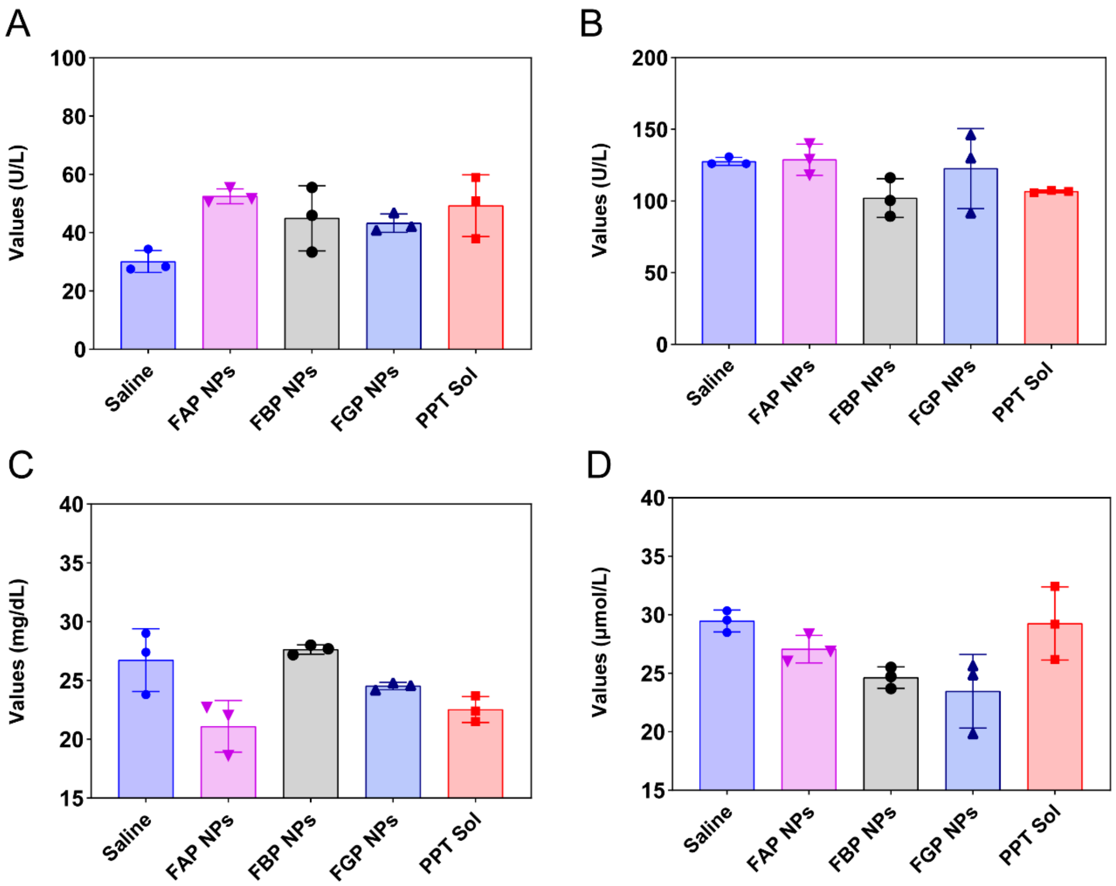

Figure S9. Blood analysis of Liver and kidney function at 11 days post administration. (n=3) (A) ALT, (B) AST, (C) BUN and (D) CREA

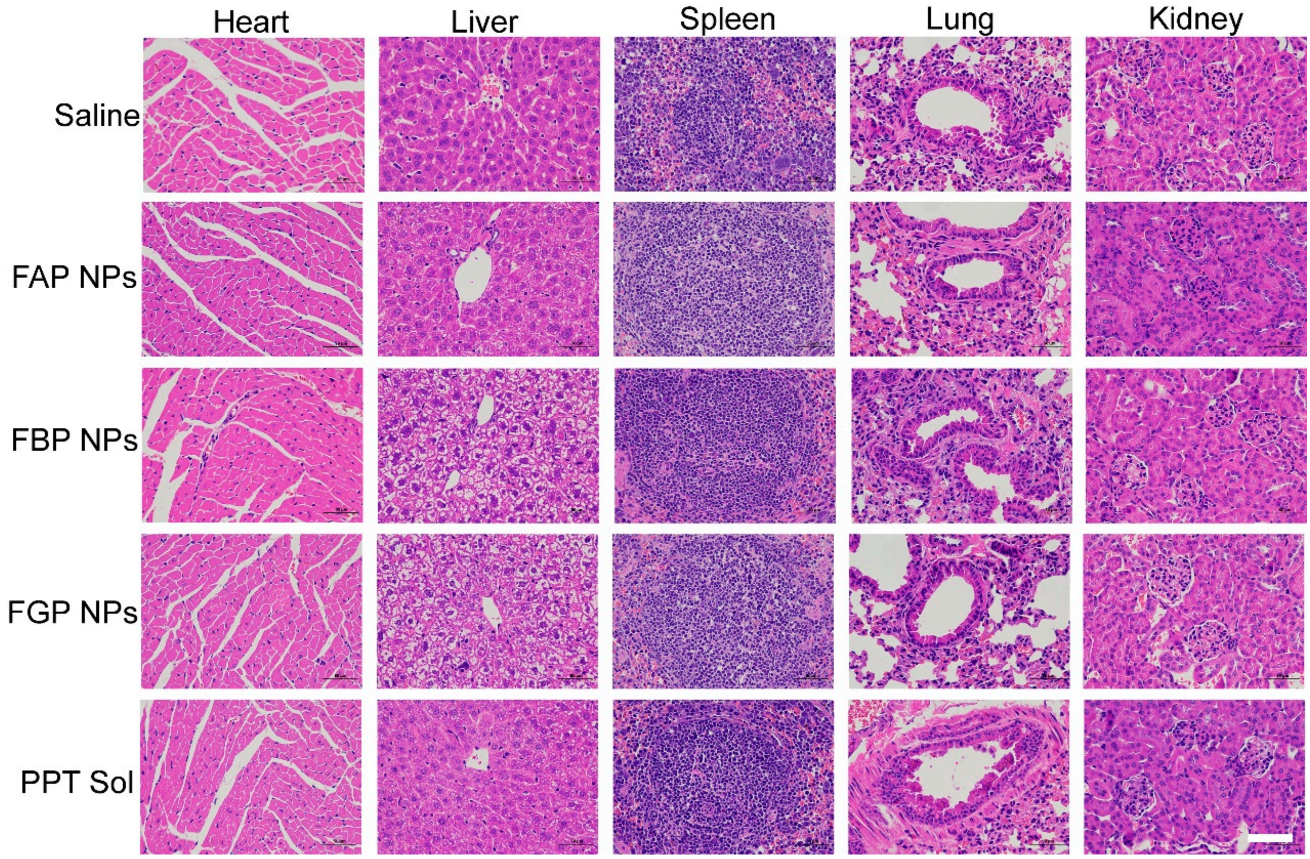

Figure S10. H&E staining images of major organ sections after the treatment. (Scale bar = 50 μm)

Table S1. Characterization of prodrug NPs.

Formulations	Size (nm)	PDI	Zeta potential (mV)	Drug loading (%)
FAP NPs	96.39±1.94	0.08±0.01	-31.0±0.8	43.8
FBP NPs	82.44±0.60	0.10±0.04	-30.4±0.6	42.2
FGP NPs	77.63±1.22	0.13±0.07	-31.9±2.4	40.8

Table S2. Cytotoxicity (IC_{50} values, ng/mL) of PPT solution and prodrug NPs.

Formulations	4T1		3T3	
	48 h	72 h	48 h	72 h
FAP NPs	33.0	57.6	474.2	283.7
FBP NPs	>1000.0	611.0	>1000.0	>1000.0
FGP NPs	695.4	470.9	>1000.0	>1000.0
PPT Sol	3.9	3.6	94.5	20.4

Table S3. Pharmacokinetic parameters of PPT solution and prodrug NPs (n=6).

Formulations	Determined	$AUC_{0\text{-}24\ h}$ (μmol/L*h)	$t_{1/2}$ (h)	$MRT_{0\text{-}24\ h}$ (h)
PPT Sol	PPT	1.67±0.24	0.30±0.07	0.31±0.07
FAP NPs	FAP	5.02±0.37	1.56±2.49	0.12±0.01
	PPT	2.31±0.34	0.37±0.04	1.13±0.23
FBP NPs	FBP	3.99±0.62	0.69±0.77	0.17±0.06
	PPT	0.15±0.05	0.67±0.17	0.75±0.27
FGP NPs	FGP	5.55±1.63	0.82±0.18	0.37±0.04
	PPT	12.51±3.20	1.03±0.50	0.12±0.02
